# Does depression increase the risk of gestational diabetes mellitus? A systematic review and meta-analysis

**DOI:** 10.12669/pjms.39.1.6845

**Published:** 2023

**Authors:** Chuanjiang Zhang, Lan Jing, Juan Wang

**Affiliations:** 1 Chuanjiang Zhang, Department of Anxiety and Depression Treatment Center, The Third Hospital of Inner Mongolia Autonomous Region, Inner Mongolia, 010010 P.R. China; 2 Lan Jing, Department of Children’s Psychological Health Center, The Third Hospital of Inner Mongolia Autonomous Region, Inner Mongolia, 010010 P.R. China; 3 Juan Wang, Department of Endocrine, The Affiliated Hospital of Inner Mongolia Medical University, Inner Mongolia, 010050 P.R. China

**Keywords:** Depression, Mental health, Diabetes mellitus, Pregnancy

## Abstract

**Objective:**

Data on the relationship between depression and gestational diabetes mellitus (GDM) is scarce and conflicting. We, hereby, aimed to review evidence if prior depression increases the risk of GDM.

**Methods:**

PubMed, Embase, CENTRAL, and Google Scholar databases were searched from inception to 11^th^ September 2021 for English language studies reporting the relationship between depression and subsequent risk of GDM.

**Results:**

Ten studies were included. Meta-analysis of data from nine studies including 127,195 patients indicated that prior depression was associated with a significantly increased risk of GDM (OR: 1.29 95% CI: 1.10, 1.52). There was no change in the significance of the results on sensitivity analysis. On subgroup analysis based on study location, we noted that the association between depression and GDM was seen only in USA-based studies with the pooled analysis of non-USA studies indicating no such relationship. Similar conflicting results were noted on subgroup analysis based on diagnostic criteria for GDM and depression.

**Conclusion:**

Our study indicates that prior depression can lead to a 29% increased risk of GDM in pregnant females. Current evidence is derived from a limited number of studies with significant heterogeneity in the timing and diagnostic criteria for depression.

## INTRODUCTION

Gestational diabetes mellitus (GDM) is a common metabolic complication which affects pregnant females. According to estimates, around six to 13% of females are affected by GDM worldwide.^1^ A recent meta-analysis including 79,064 Chinese participants has shown that the pooled prevalence of GDM in mainland China is 14.8% ranging from 12.8 to 16.7%.^2^ Research also suggests that the prevalence of GDM is increasing gradually and the disease could lead to a significant financial burden on the healthcare system in the near future.^3^

While there have been recent debates on the value of detecting and managing GDM, there is strong evidence that suggests that GDM leads to several maternal and fetal complications like preeclampsia, premature birth, type2 diabetes mellitus, fetal malformations, and perinatal mortality.^4^ Considering such adverse outcomes, it is imperative for clinicians to identify early risk factors for development of GDM in order to control its incidence.

In recent years, several studies have identified a link between depression and incident diabetes in the general population.^5^ Interestingly, the tendency of the association has been bidirectional with depression increasing the risk of diabetes and diabetes in turn increasing the risk of subsequent depression.^6^ While the exact cause of such relationship is unclear, it may stem from the fact that both depression and diabetes having a common pathophysiological mechanism contributed by increased oxidative stress and inflammation.^7^

Since depression is relatively common amongst pregnant females^8^, it is essential to understand if similar heightened risk of GDM exists in pregnant patients with prior depression. In 2019, Arafa et al^9^ examined this by means of a meta-analysis. However, their review could include just five studies. Furthermore, there has been wide variation in the results of individual studies, with one^10^ demonstrating no association between depression and subsequent GDM while another^11^ reporting opposing results. Also, with publication of new studies in the past two years, there is a need for updated evidence to gauge the relationship between depression and the risk of GDM. Therefore, the aim of the current study was to conduct a systematic literature search of cohort studies and pool data to assess if prior depression increases the risk of GDM.

## METHODS

This systematic review and meta-analysis were carried out according to the guidelines of the PRISMA statement (Preferred Reporting Items for Systematic Reviews and Meta-analyses).^12^ The PROSPERO registration number of the study is CRD42021272248. Initially, the protocol was registered to assess the relationship between anxiety and depression and risk of GDM. However, since very few studies were found to report the relationship between anxiety and GDM, the protocol was amended to include depression only.

### Literature search:

Two reviewers independently searched the electronic databases of PubMed, Embase, CENTRAL, and Google Scholar for relevant articles. The search limits were set from the inception of the above-mentioned databases to 11^th^ September 2021. The search terms used were: “depression”, “depressive symptoms”, “gestational diabetes”, and “pregnancy diabetes”. Details of the search strategy common to all databases are presented in Supplementary Table-I. The primary search results were assessed initially by their titles and abstracts to identify citations requiring full-text analysis. The full texts of the articles were reviewed by the two reviewers independently based on the inclusion and exclusion criteria. Any disagreements were resolved by discussion with the third reviewer. We also carried out manual scoping of the bibliography in included studies for any additional articles.

**supplementary Table-I:** Search strategy.

Search number	Query	Search Details
1	(depression) AND (gestational diabetes)	("depressed"[All Fields] OR "depression"[MeSH Terms] OR "depression"[All Fields] OR "depressions"[All Fields] OR "depression s"[All Fields] OR "depressive disorder"[MeSH Terms] OR ("depressive"[All Fields] AND "disorder"[All Fields]) OR "depressive disorder"[All Fields] OR "depressivity"[All Fields] OR "depressive"[All Fields] OR "depressively"[All Fields] OR "depressiveness"[All Fields] OR "depressives"[All Fields]) AND ("diabetes, gestational"[MeSH Terms] OR ("diabetes"[All Fields] AND "gestational"[All Fields]) OR "gestational diabetes"[All Fields] OR ("gestational"[All Fields] AND "diabetes"[All Fields]))
2	(depression) AND (pregnancy diabetes)	("depressed"[All Fields] OR "depression"[MeSH Terms] OR "depression"[All Fields] OR "depressions"[All Fields] OR "depression s"[All Fields] OR "depressive disorder"[MeSH Terms] OR ("depressive"[All Fields] AND "disorder"[All Fields]) OR "depressive disorder"[All Fields] OR "depressivity"[All Fields] OR "depressive"[All Fields] OR "depressively"[All Fields] OR "depressiveness"[All Fields] OR "depressives"[All Fields]) AND ("pregnancy in diabetics"[MeSH Terms] OR ("pregnancy"[All Fields] AND "diabetics"[All Fields]) OR "pregnancy in diabetics"[All Fields] OR ("pregnancy"[All Fields] AND "diabetes"[All Fields]) OR "pregnancy diabetes"[All Fields])
3	(depressive symptoms) AND (gestational diabetes)	("depression"[MeSH Terms] OR "depression"[All Fields] OR ("depressive"[All Fields] AND "symptoms"[All Fields]) OR "depressive symptoms"[All Fields]) AND ("diabetes, gestational"[MeSH Terms] OR ("diabetes"[All Fields] AND "gestational"[All Fields]) OR "gestational diabetes"[All Fields] OR ("gestational"[All Fields] AND "diabetes"[All Fields]))

### Inclusion criteria:

- All types of prospective or retrospective cohort studies conducted on pregnant females.
- Studies were to report a temporal analysis between depression and subsequent risk of GDM.
- Studies were to report odds ratios (OR) or risk ratios (RR) with 95% confidence intervals (CI) of the association.

### Exclusion criteria:

- Studies assessing the concomitant risk of depression and GDM or assessing the risk of postnatal depression due to GDM
- Cross-sectional studies or case-control studies using a convenience sample.
- Non-English language studies, abstracts, editorials, review articles, and case reports. Five studies with a repeated or overlapping sample.

### Data extraction and Risk of bias assessment:

A data extraction sheet was used by two reviewers to extract relevant data from the studies. The details extracted included first author, publication year, study type, study location, sample size, type of stroke, mean age, body mass index (BMI), and diagnostic criteria for GDM and depression, the timing of diagnosis of depression, number of GDM cases, OR/RR of the association, and factors adjusted during the calculation of OR/RR. The outcome of the study was risk of GDM due to prior depression. The diagnostic criteria for GDM and depression were not defined and definitions of individual studies were used.

The methodological quality of studies was assessed using the Newcastle-Ottawa Scale (NOS).^13^ It was conducted by two authors independent of each other. Any disagreements were solved by a discussion. Studies were assessed for selection of study population, comparability, and outcomes, with each domain being awarded a maximum of four, two, and three points respectively.

### Statistical analysis:

“Review Manager” (Rev Man, version 5.3; Nordic Cochrane Centre [Cochrane Collaboration], Copenhagen, Denmark; 2014) was used for the meta-analysis. We extracted OR/RR with a 95% CI of the association between depression and GDM from the included studies. These were then pooled to calculate the total effect size as OR with 95% CI. The random-effects model was used. Heterogeneity was assessed using the I^2^ statistic.

We visually inspected funnel plots to assess publication bias. We also conducted a sensitivity analysis wherein individual studies were excluded one at a time and the effect size was recalculated. Sub-group analysis was also carried out based on the location of studies, study type, diagnostic criteria of GDM, and depression.

## RESULTS

**Fig.1:**
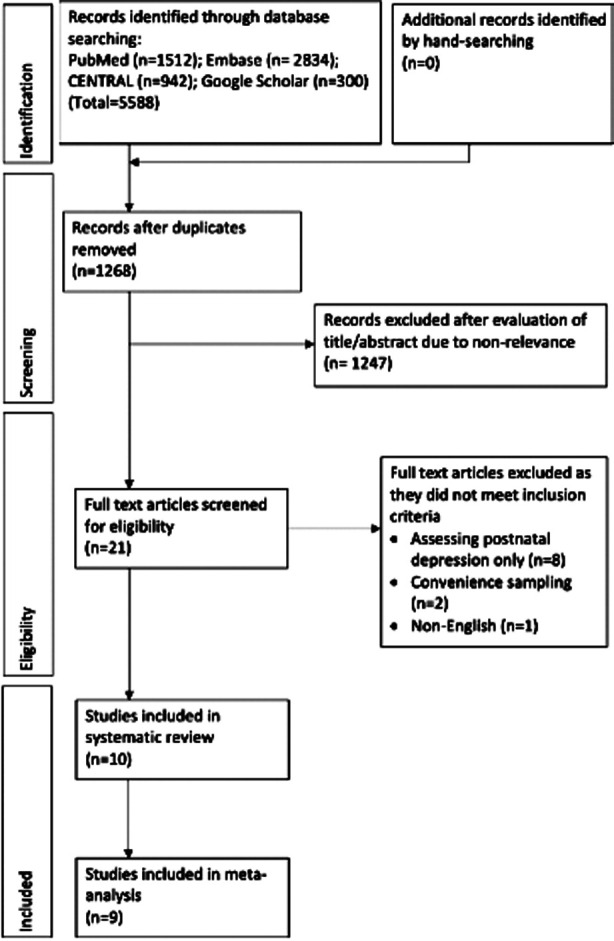
Study flow chart.

A total of 10 studies met the inclusion criteria and were included in this review.^10^,^11^,^14^–^21^ Fig.1 Details of these studies are presented in Table-I. Three were retrospective while six were prospective cohort studies. One study^10^ which was a secondary analysis of a randomized controlled trial was also included after consultation amongst the reviewers as it presented a temporal association between depression and subsequent risk of GDM.

**Table-I:** Characteristics of included studies

Study	Location	Type	Sample size	Mean age	Pre-pregnancy BMI (kg/m^2^)	BMI ≥30 (%)	Diagnosis of GDM	Percentage of GDM cases	Diagnosis of Depression/ Anxiety	Depression/ anxiety diagnosis timing	Adjusted confounders	NOS score
Bowers 2013[21]	USA	R	111,952	NR	NR	NR	ICD-9	NR	ICD codes	Prior to GDM	Age, race/ethnicity, parity, BMI	8
Dahlen 2015[20]	Australia	R	3092	29.2± 5.5	NR	26	NR	10.7	EPDS ≥ 13	Prior to GDM	Age, race/ethnicity, parity, BMI, smoking	7
Wilson 2015[11]	USA	R	3655	27.5± NR	25.1± NR	NR	Self-report	5.3	Self-report	Before pregnancy	Age, race/ethnicity, BMI, income, smoking	6
Hinkle 2016[18]	USA	P	2477	28.1± 5.5	25.5± 2.5	NR	OGTT	4.2	EPDS ≥ 10	1^st^ trimester	Age, race/ethnicity, BMI, education, marital status	8
Morrison 2016[19]	USA	P	1021	25.8± NR	28.5± NR	NR	NR	8.1	CES-D ≥ 10	1^st^ trimester	Age, BMI, education, income, marital status, previous miscarriage, pregnancy weight gain	7
Larrabure-Torrealva 2018[17]	Peru	P	1300	28.9± 6.1	NR	11.5	OGTT	15.8	PHQ-9 ≥ 10	Prior to GDM	Age, race/ethnicity, marital status, education, DMI, family history of DM, comorbidities, income	8
Molyneaux 2018[10]	UK	RCT*	1555	30.5± 5.5	NR	100	OGTT	28.2	EPDS ≥ 13	1^st^ trimester	Age, BMI, race/ethnicity, marital status, education, income, index of multiple deprivation, parity and study centre	8
Wilson 2020[16]	UK	P	13539	27.3± 5.6	26± 5.7	NR	OGTT	7.6	ICD codes	Before pregnancy	Age, education, race/ethnicity, pre-eclampsia, gestational hypertension	8
Minschart 2021[15]	Belgium	P	1843	NR	NR	12.5	OGTT	12.5	CES-D ≥ 16	Prior to GDM	Age, race/ethnicity, education, smoking, BMI	8
Versteegen 2021[14]	USA	P	300	28.5± 5.5	29± 8.8	NR	OGTT	8.3	EPDS > 10	Prior to GDM	Age, BMI	8

BMI, body mass index; EPDS, Edinburgh Postnatal Depression Scale; GDM, gestational diabetes mellitus; ICD, International Classification of Diseases; NR, not reported; OGTT, oral glucose tolerance test; P, prospective cohort; R, retrospective cohort; RCT, randomized controlled trial; PHQ-9, Patient Health Questionnaire-9; CES-D, Center for Epidemiologic Studies–Depression questionnaire. *secondary analysis of the RCT.

The sample size of the studies ranged from 300 to 111952 patients. In six studies, an oral glucose tolerance test (OGTT) was used to diagnose GDM while two studies presented no information on the diagnostic criteria. The percentage of GDM patients in the studies ranged from 4.2 to 28.2%. In three studies, depression was diagnosed in the first trimester of pregnancy and in two studies it was diagnosed before pregnancy. In the remaining studies, there was a lack of clarity on the exact time of diagnosis of depression.

The criteria of diagnosis of depression were also variable. All included studies reported a multivariable-adjusted effect size but the factors adjusted in the multivariate analysis were variable. Of the included studies, Wilson et al^16^ reported the combined impact of anxiety and depression on the risk of GDM. The remaining studies assessed only the impact of prior depression on the risk of GDM. On evaluation of study quality, majority of studies received a score of eight on NOS while one study scored seven, indicating moderate risk of bias. The study of Wilson et al^11^ was considered to have high risk of bias with a score of six.

### Analysis:

**Fig.2:**
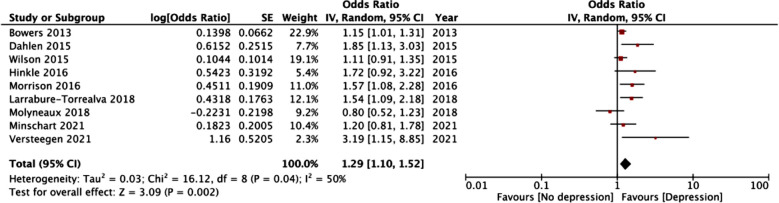
Meta-analysis of prior depression and risk of GDM.

Meta-analysis of data from nine studies including 127,195 patients indicated that prior depression was associated with a significantly increased risk of GDM (OR: 1.29 95% CI: 1.10, 1.52 I^2^=50% p=0.04) (Fig.2). We failed to notice any publication bias. Fig.2 Results of sensitivity analysis are presented in Table-II. It can be noted that the results did not change in statistical significance at the exclusion of any of the included studies.

**Table-II:** Results of sensitivity analysis.

Study	Resultant Odds ratios
Bowers 2013[21]	1.36 95% CI: 1.10, 1.68 I^2^=53% p=0.005
Dahlen 2015[20]	1.25 95% CI: 1.06, 1.46 I^2^=47% p=0.007
Wilson 2015[11]	1.35 95% CI: 1.11, 1.66 I^2^=54% p=0.003
Hinkle 2016[18]	1.27 95% CI: 1.07, 1.50 I^2^=53% p=0.005
Morrison 2016[19]	1.26 95% CI: 1.06, 1.49 I^2^=50% p=0.008
Larrabure-Torrealva 2018[17]	1.26 95% CI: 1.06, 1.50 I^2^=50% p=0.009
Molyneaux 2018[10]	1.34 95% CI: 1.14, 1.56 I^2^=44% p=0.0003
Minschart 2021[15]	1.31 95% CI: 1.09, 1.57 I^2^=57% p=0.004
Versteegen 2021[14]	1.25 95% CI: 1.08, 1.46 I^2^=45% p=0.003

CI, confidence intervals.

**Fig.3:**
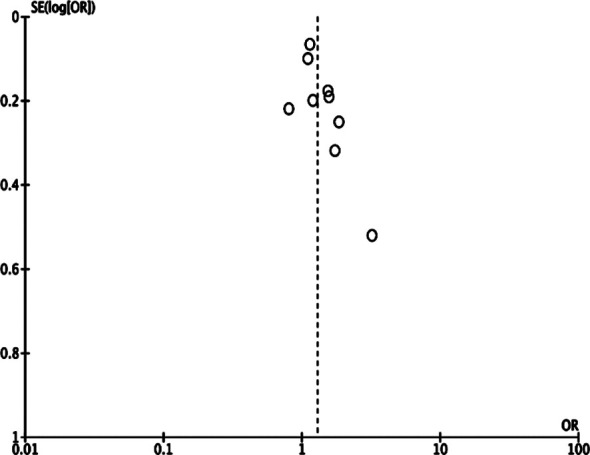
Funnel plot for the meta-analysis of prior depression and risk of GDM.

Results of subgroup analysis are presented in Table-III. We noted that the association between depression and GDM was seen only in USA-based studies. The results were, however, statistically significant for both retrospective and prospective studies. On subgroup analysis based on the diagnosis of GDM, we noted no association between depression and GDM when the latter was diagnosed using OGTT. But the association was significant in non-OGTT studies. In the four studies diagnosing depression using the Edinburgh Postnatal Depression Scale (EPDS), we noted no relationship between depression and GDM but a statistically significant association was seen for the remaining groups.

**Table-III:** Subgroup analysis.

Variable	Groups	Number of studies	Odds ratio
Study location	USA	5	1.28 95% CI: 1.06, 1.55 I^2^=48% p=0.01
	Non-USA	4	1.28 95% CI: 0.92, 1.79 I^2^=62% p=0.15
Study type	Prospective	6	1.38 95% CI: 1.05, 1.82 I^2^=52% p=0.02
	Retrospective	3	1.19 95% CI: 1.00, 1.42 I^2^=45% p=0.04
Diagnosis of GDM	OGTT	5	1.35 95% CI: 0.96, 1.90 I^2^=59% p=0.09
	Non-OGTT	4	1.25 95% CI: 1.05, 1.49 I^2^=49% p=0.01
Diagnosis of depression	EPDS	4	1.54 95% CI: 0.89, 2.69 I^2^=72% p=0.13
	CES-D	2	1.38 95% CI: 1.05, 1.81 I^2^=0% p=0.02
	Others	3	1.18 95% CI: 1.03, 1.35 I^2^=28% p=0.01

EPDS, Edinburgh Postnatal Depression Scale; GDM, gestational diabetes mellitus; OGTT, oral glucose tolerance test; CES-D, Center for Epidemiologic Studies–Depression questionnaire.

Since one study was not included in the meta-analysis, its results are hereby presented descriptively. Wilson et al^16^ in their study reported that anxiety or depression before pregnancy was not associated with an increased risk of GDM (RR: 0.96; 95% CI 0.80,1.15).

## DISCUSSION

Depression during prenatal and early pregnancy is common affecting around 6.5-20% of females.^8^ Antenatal depression leads to poor maternal-fetal attachment, and such patients are at an increased risk of postpartum depression which can significantly impact the mental health and social relationships of the entire household.^22^

Depression during pregnancy has also been associated with a heightened risk of preterm birth, low birth weight, and cesarean deliveries.^23^ Such increased awareness of the physical-mental health interface has led to an acknowledgment of the bi-directional relationship between depression and diabetes mellitus.^24^ Indeed, a meta-analysis has demonstrated that depression is associated with an increased risk of incident diabetes in the general population (RR: 1.38 95% CI: 1.23-1.55).^5^ On the other hand, around 30% of diabetics have symptoms of depression which can hamper disease management and glycemic control.^25^

Despite several studies demonstrating a significant relationship between depression and diabetes, research linking depression with GDM has been scarce. However, there is evidence of the bi-directional relationship between depression and GDM too. Arafa et al^26^ have demonstrated a significantly increased risk of postpartum depression in women with GDM. In another review by the same authors, prior depression was associated with a significantly increased risk of GDM as well.^9^ However, an important limitation of this review was that it could include only five studies. To provide strengthened evidence to practicing clinicians, we hereby aimed to update their study. Similar to the previous review, we also noted a statistically significant increased risk of GDM in patients with prior depression.

It can be noted from the forest plot that four of the included studies noted no relationship between depression and GDM.^10^,^11^,^15^,^18^ However, in two studies the lower levels of the 95% CI were close to one (0.91 for Wilson et al^11^ and 0.92 for Hinkle et al^18^), indicating that there may be a link between the two diseases. Our results are also supported by another review by Wilson et al^27^ which noted an increased prevalence of depressive symptoms in women around the time of diagnosis of GDM.

The mechanism by which depression can lead to GDM is, however, unclear. Research suggests that depression and diabetes may have common pathophysiological mechanisms involving over activation of innate immunity and the hypothalamus-pituitary–adrenal axis which causes increased production of cytokines and stress hormones. These cytokines can directly affect the brain and cause depressive symptoms. The inflammatory response and overproduction of stress hormones can also affect the pancreatic cells leading to insulin resistance.^7^,^24^ Furthermore, the presence of depression is linked to poor lifestyle habits like physical inactivity, unhealthy diet, and behavioral issues which can increase the risk of diabetes.^24^

The heterogeneity in our meta-analysis was 50% which was classified as “medium”. This was however expected owing to several methodological variations amongst the included studies. On subgroup analysis, our results indicated that the association between depression and GDM was significant only for USA-based studies but not for non-USA-based studies. Such variation is difficult to comprehend as the majority of the countries in the non-USA group were also developed countries and the question of difference in the quality of healthcare should not arise.

Nevertheless, the analysis of non-USA-based studies demonstrated a lower 95% CI of 0.92 and the non-significant results could be partly attributed to the lower number of participants in this sub-group. Importantly, we noted that the association between depression and GDM was not statistically significant when GDM was diagnosed using the gold-standard OGTT but the outcomes were significant for the remaining studies.

This is of particular concern as non-standardized tests can lead to a false diagnosis of GDM^28^ and thereby impact the link between depression and GDM. Similarly, there was also marked variation between the studies on the diagnostic criteria for depression, and our sub-group analysis presented conflicting results. Essentially, there is a need for further prospective studies using standardized diagnostic criteria for both GDM and depression to understand the link between the two diseases.

Another important factor to consider while interpreting our results is that the risk of GDM can be influenced by several confounders. Indeed, maternal obesity has been identified as one of the most important risk factors for GDM.^29^ Maternal BMI was adjusted in the multivariate analysis by the majority of the included studies thereby strengthening the fact that depression may independently increase the risk of GDM. However, Hinkle et al^18^ in their study noted that the presence of depression increased the risk of GDM only in non-obese women and no such association was noted in obese females.

The study of Molyneaux et al^10^ which included only obese women also noted no increased risk of GDM with prior depression in their cohort (OR: 0.80 95% CI: 0.52, 1.23). One reason for the non-significant association could be that obese women, in general, have a baseline higher risk of GDM which is not overtly changed by depression. However, this needs to be clarified by future studies.

### Limitations:

Despite being an updated review, the number of studies in the meta-analysis was just nine. Secondly, there was significant methodological variation amongst the studies with regards to important factors like the timing of diagnosis of depression, the diagnostic criteria for GDM and depression, etc. Even amongst studies using the same diagnostic tool for depression, the cutoff for diagnosis varied. Such variations have important implications on the validity of our results and could significantly skew the outcomes. Thirdly, the factors adjusted in the multivariate analysis were not exactly coherent amongst the included studies. Missed known and other unknown confounders may have over or underestimated the ORs/RRs.

## CONCLUSIONS

The results of our study indicate that prior depression can lead to a 29% increased risk of GDM in pregnant females. Current evidence is derived from a limited number of studies with significant heterogeneity in the timing and diagnostic criteria for depression. Future studies are needed to supplement our conclusions.

### Authors’ contributions:

**CZ and LJ** conceived and designed the study.

**CJ, LJ and JW** collected the data and performed the analysis.

**CZ and LJ** were involved in the writing of the manuscript and is responsible for the integrity of the study.

All authors have read and approved the final manuscript.
